# Multiple Salivary Cortisol Measurements Are a Useful Tool to Optimize Metyrapone Treatment in Patients with Cushing’s Syndromes Treatment: Case Presentations

**DOI:** 10.3389/fendo.2017.00375

**Published:** 2018-01-11

**Authors:** Kenichi Yoshida, Hidenori Fukuoka, Yukiko Odake, Shinsuke Nakajima, Mariko Tachibana, Jun Ito, Yusei Hosokawa, Tomoko Yamada, Hiroshi Miura, Natsu Suematsu, Ryusaku Matsumoto, Hironori Bando, Kentaro Suda, Hitoshi Nishizawa, Genzo Iguchi, Wataru Ogawa, Yutaka Takahashi

**Affiliations:** ^1^Division of Diabetes and Endocrinology, Kobe University Graduate School of Medicine, Kobe, Japan; ^2^Division of Diabetes and Endocrinology, Kobe University Hospital, Kobe, Japan

**Keywords:** Cushing’s syndrome, salivary cortisol, metyrapone, diurnal rhythm, monitoring marker

## Abstract

Measuring salivary cortisol is both convenient and non-invasive for patients; however, its usefulness as a marker for monitoring medical therapy has not yet been established. The aim of this study was to assess the utility of multiple salivary cortisol measurements in patients with Cushing’s syndrome (CS) during medical therapy. Six patients with CS (three with cortisol-secreting adrenocortical adenoma and three with ACTH-secreting pituitary adenoma) were recruited. Samples for morning serum cortisol, urinary free cortisol (UFC), and multiple salivary cortisol levels were collected before and during metyrapone treatment. The area under the curve (AUC) and mean value (MV) of daily salivary cortisol levels were calculated. In five out of six patients, UFC were normalized; however, multiple salivary cortisol measurements revealed an impaired diurnal cortisol rhythm in these patients. To verify the usefulness of multiple salivary cortisol measurements, we performed a prospective case study of a patient in whom the excess secretion of cortisol was not controlled (UFC 211 μg/day) with 2,250 mg/day in four divided doses of metyrapone. Multiple measurements of salivary cortisol revealed that cortisol levels elevated before the next administration. Accordingly, we shortened the interval by increasing the number of administration from four to five times per day, with a slight increment of daily dose of 2,500 mg. These optimizations resulted in a drastic improvement of diurnal pattern as well as UFC level (101 μg/day). Changes in both the MV and AUC of salivary cortisol levels were more correlated with those in UFC levels (Correlation coefficient 0.75, *p* = 0.007, and 0.70, *p* = 0.017) than those in the morning serum cortisol levels (0.42, *p* = 0.200), indicating that multiple salivary cortisol measurements reflect more precisely the excess secretion of cortisol. Our preliminary data suggest that multiple salivary cortisol measurements can be a useful tool to visualize the diurnal cortisol rhythm and to determine the dose and timing of metyrapone during the treatment in patients with CS.

## Introduction

Cushing’s syndrome (CS) is associated with high mortality and serious morbidities such as infections, cardiovascular disease, stroke, and thromboembolism (1, 2). Thus, prompt treatment for hypercortisolism is essential. To control the excess of cortisol, adrenal steroidogenesis inhibitors such as metyrapone and ketoconazole have commonly been used (3, 4). Recently, the novel steroidogenesis inhibitor LCI699, an inhibitor of 11β-hydroxylase, has been developed (5, 6). Using these drugs, it is important to not only suppress cortisol secretion but also avoid adrenal insufficiency and reconstitute the circadian rhythm. However, there have been few useful biomarkers for evaluation of cortisol activity during medical therapy. While undergoing treatment with steroidogenesis inhibitors, early morning serum cortisol, average daily serum cortisol, and daily urinary free cortisol (UFC) have each been suggested as markers for treatment monitoring (7, 8). However, adjusting the dose and timing of drug administration remains challenging.

Collection of samples for salivary cortisol management is convenient for patients and is feasible in outpatient clinics (9, 10). The utility of salivary cortisol measurements for the diagnosis and prediction of the risk of the recurrence of CS has been well established (11). However, there have been few reports that demonstrate whether the measurement of salivary cortisol could be a valuable marker for monitoring medical therapy. The aim of this study was to determine the utility of salivary cortisol measurements for the optimization of dosage and timing of medical therapy in patients with CS.

## Materials and Methods

### Patients

This protocol was approved by the Kobe University Hospital ethical committee (IRB No. 1351). Written informed consent was obtained from all patients. Six patients with CS (three with cortisol-secreting adrenocortical adenoma (A); *n* = 3 and three with ACTH-secreting pituitary adenoma (P); *n* = 3, Table 1) who had been hospitalized at Kobe University Hospital during the period from 2013 to 2016 were recruited in this study. All patients presented with symptoms of overt CS including moon faces, central obesity, and buffalo humps. The diagnosis of CS was based on general diagnostic criteria as previously described (12, 13). This study was performed during medical therapy to control hypercortisolism before surgery in most of the patients. Five of the six patients received metyrapone treatment for preoperative control of hypercortisolemia. The remaining patient (No. 4) received metyrapone for medical control of a residual ACTH-secreting pituitary adenoma. In patients who underwent surgery, the diagnosis of cortisol-secreting adrenocortical adenoma or ACTH-secreting pituitary adenoma was histologically confirmed.

**Table 1:** Clinical characteristics of patients with Cushing’s syndrome.

Patient no.	1	2	3	4	5	6
Cause of CS	A	A	A	P	P	P
Age (years)	48	43	64	35	55	22
M/F	F	F	F	F	M	F
ACTH (pg/mL)	<5.0	<5.0	<5.0	32.6	179	79.9
Serum cortisol (μg/dL)	21.2	21	19.1	19.2	38.6	29.4
UFC (μg/day)	260	97.3	119.5	85.6	1,980	329.3
L-DST cortisol (μg/dL)	18.1	18.7	15.7	14	38.6	25.3

*A, cortisol-secreting adrenocortical adenoma; P, ACTH-secreting pituitary adenoma; L-DST, low dose dexamethasone suppression test*.

### Saliva Collection

Subjects rinsed mouth thoroughly with water 10 min before the sample is collected. They tilted the head forward, allowing the saliva to pool on the floor of the mouth, then passed the saliva into a polypropylene tube. Samples were centrifuged (1,500 × *g*, 15 min), the supernatant transferred into new tubes, and stored at −20°C until measurement. Sample collection was not set within 60 min after eating a major meal.

### Measurement Protocol

The salivary cortisol collection was performed five times in a day: at 6:00 (wake up time), 8:00 (before breakfast), 12:00 (before lunch), 18:00 (before dinner), and 22:00 (before sleep). A daily mean value (MV) and daily area under the curve (AUC) of salivary cortisol levels were calculated (MV: average of five measurements of salivary cortisol levels and AUC: integral of five measurements of salivary cortisol levels).

The samples were collected before metyrapone treatment, during metyrapone treatment, and when the dose of metyrapone was increased. The date of sampling was set for 2 days after the start or increase in the dose of metyrapone. The initial dose of metyrapone was 500–750 mg per day, and the maintenance dose was 500–2,000 mg per day. The administration timing was 14:00 and 22:00 for patients receiving the drug twice per day (500 mg per day); 6:00, 14:00, and 22:00 for patients receiving the drug three times per day (750–1,250 mg per day); and patients receiving the drug at 6:00, 12:00, 18:00, and 24:00 for four times per day (1,000–2,000 mg per day). The dosage and timing for each patient were determined based on the evaluation of the morning serum cortisol levels, the UFC levels, and the well-being of each patient. The target levels of morning serum cortisol and UFC were below the upper normal limit. Salivary cortisol measurements were performed, and its utility was analyzed retrospectively. In patient No. 6, salivary samples before treatment were not obtained. Therefore, only the changes in MV and AUC during treatment were analyzed in this patient.

Patient No. 4 was hospitalized again due to worsening of hypercortisolemia 1 year after the first hospitalization. To test whether the salivary cortisol is useful, we collected and measured multiple salivary samples from this patient during the treatment and adjusted the dose and timing of metyrapone administration as a prospective case study.

### Hormone Assays

The serum cortisol levels were measured by an enzyme immunoassay (reference range: 6.4–21.0 µg/dL, EIA; TOSOH, Tokyo, Japan), and the daily UFC levels were measured by radioimmunoassay (reference range: 11.2–80.3 μg/day, RIA; TFB, Tokyo, Japan). Salivary cortisol levels were measured by enzyme immunoassay (Salimetrics Inc., State College, PA, USA).

### Statistical Analysis

All statistical analyses were performed using SAS 11.2 (Statistical Analysis Software release 11.2; SAS Institute Inc., Cary, NC, USA). Data are shown as mean ± SD. The paired *t*-test was used to compare the values of mean from two related samples. The correlation between non-parametric data was assessed with Spearman’s rank correlation. The cutoff for statistical significance was set at *p* < 0.05.

## Results

### Clinical Characteristics of Patients with CS

The clinical characteristics of the patients are described in Table 1. The patient ages ranged from 22 to 64 years. Five of six were female. The basal ACTH level was undetectable in all of the patients with an adrenocortical adenoma and was 32.6–179.0 pg/mL in the patients with an ACTH-secreting pituitary adenoma. The range of morning serum cortisol levels and UFC levels were 19.2–38.6 µg/dL and 97.3–1,980.0 μg/day, respectively. The serum cortisol level after a low-dose dexamethasone suppression test was 14.0–38.6 µg/dL. These data were consistent with an active state of CS.

### Changes in Morning Serum Cortisol and UFC Levels before and after Initiation of Metyrapone Treatment

**Figure 1:**
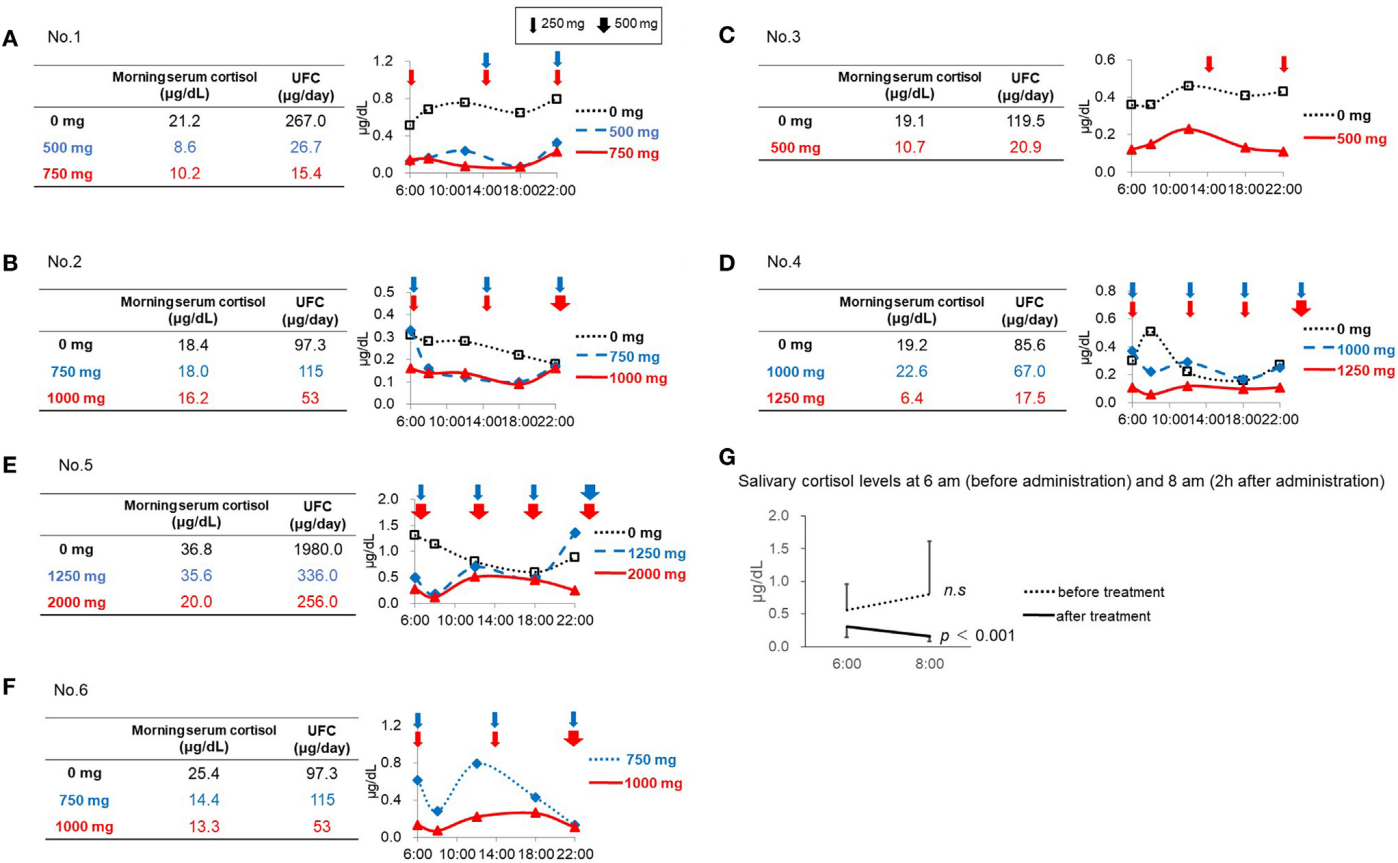
Salivary cortisol levels before and after initiation of metyrapone treatment. **(A)** Patient No. 1. **(B)** Patient No. 2. **(C)** Patient No. 3. **(D)** Patient No. 4. **(E)** Patient No. 5. **(F)** Patient No. 6. **(G)** Salivary cortisol levels at 6:00 a.m. (before administration) and 8:00 a.m. (2 h after administration).

Both morning serum cortisol and UFC levels were decreased within the normal range after initiation of treatment in all patients except for Patient No. 5 (Figure 1). Patient No. 5 was referred to neurosurgery before hormonal management achieved target levels.

### Retrospective Assessment of Salivary Cortisol Levels in Each Case

In Patient No. 1, both the UFC and morning serum cortisol levels decreased after initiation of metyrapone treatment in a dose-dependent manner (Figure 1A). However, multiple measurements of salivary cortisol levels revealed relatively lower cortisol levels in the morning and higher levels in the daytime and night, indicating that the diurnal cortisol rhythm was still impaired in this patient (Figure 1A). Similar results were observed in Patients No. 3 and 4 (Figures 1C,D). In Patient No. 2 (Figure 1B), the serum cortisol and UFC levels did not change with 750 mg of metyrapone, while the MV and AUC of salivary cortisol levels decreased. Furthermore, when the dose was increased at night by 500 mg, morning cortisol levels were suppressed in both serum and saliva. However, the night salivary cortisol did not decrease. These results suggest that not only the dose but also the timing should be considered to reconstitute the diurnal rhythm.

In Patient No. 5, despite a marked reduction in UFC levels, the morning serum cortisol levels did not change with 1,250 mg of metyrapone. We then increased the dose to 2,000 mg, resulting in decrease in both morning serum cortisol and UFC levels, though still not up to target levels. This suggests that a further increase in the dose might be necessary. Salivary cortisol levels revealed that, even with the dose of metyrapone 2,000 mg, the CS remained uncontrolled, and obvious impairment of the diurnal cortisol rhythm remained (Figure 1E).

In Patient No. 6, UFC levels and morning serum cortisol levels were both normalized after metyrapone treatment. However, the diurnal rhythm was not fully reconstituted (Figure 1F).

### Prospective Case Study Based on Salivary Cortisol Levels

**Figure 2:**
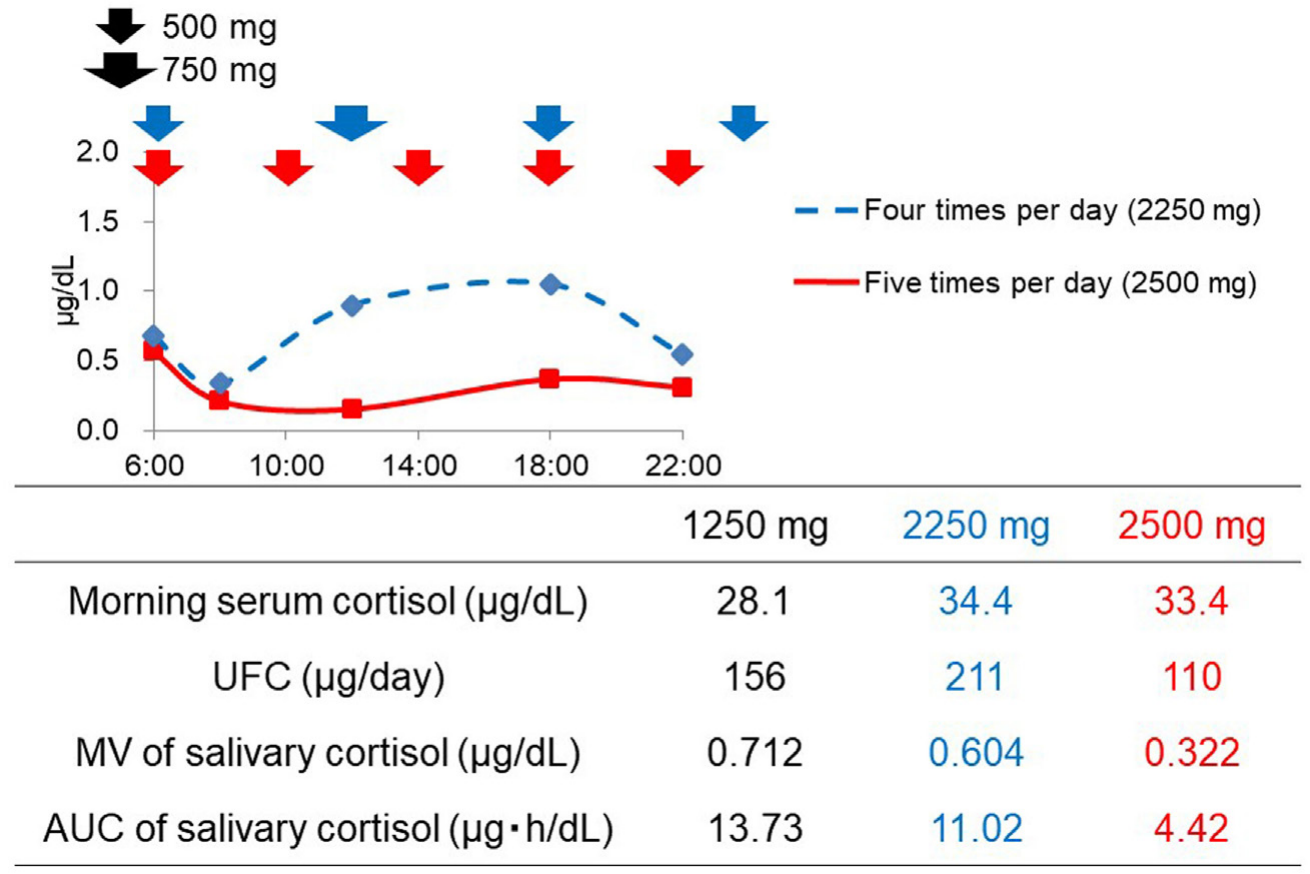
Changes in the diurnal salivary cortisol pattern after the optimization of timing of metyrapone administration in Patient No. 4.

Patient No. 4 was readmitted to the hospital 1 year after the first admission due to the worsening in hypercortisolemia despite a dose of 1,250 mg of metyrapone. During the second admission, although the dose of metyrapone was increased to 2,250 mg, the UFC level rather increased (Figure 2). According to the diurnal salivary cortisol level, it was observed that when administered four times a day, the efficacy did not last until next administration. Therefore, we shortened the dosing interval and increased the frequency from four (6:00, 12:00, 18:00, and 24:00) to five times per day (6:00, 10:00, 14:00, 18:00, and 22:00). As a result, the diurnal salivary cortisol pattern was obviously improved, despite the slight increase in the total daily dose (Figure 2). The UFC level also decreased from 211 to 110 μg/day, indicating that appropriate optimization had been obtained. Furthermore, insomnia due to hypercortisolemia was markedly improved. In this series, there were no adverse events that were related to metyrapone treatment.

### Diurnal Variation of Salivary Cortisol before and after Initiation of Treatment

Both the MV and AUC of salivary cortisol levels decreased after initiation of metyrapone treatment (Table 2). Metyrapone treatment suppressed diurnal salivary cortisol levels in a dose-dependent manner (Figures 1A–F). The effect of metyrapone on salivary cortisol levels was observed 2 h after administration of the drug (Figure 1G). Changes in both the MV and AUC of salivary cortisol levels were significantly correlated with changes in UFC levels (*r*_s_ = 0.75 and 0.70, *p* = 0.007 and 0.016, respectively), whereas changes in the morning serum cortisol levels were not (*r*_s_ = 0.42, *p* = 0.200; Table 3). On the other hand, changes in both the MV and AUC of salivary cortisol levels were not correlated with those in midnight salivary cortisol levels (Table 4).

**Table 2:** Salivary cortisol levels before and after initiation of metyrapone treatment.

	MV of salivary cortisol (μg/dL)	Area under the curve (AUC) of salivary cortisol (μg · h/dL)
**(a) No. 1**		
0 mg	0.64	11.2
500 mg	0.19	2.9
750 mg	0.16	2.7
**(b) No. 2**		
0 mg	0.25	4.0
750 mg	0.18	2.3
1,000 mg	0.14	2.0
**(c) No. 3**		
0 mg	0.40	6.7
500 mg	0.15	2.6
**(d) No. 4**		
0 mg	0.29	4.3
1,000 mg	0.28	4.1
1,250 mg	0.10	1.6
**(e) No. 5**		
0 mg	0.95	13.5
1,250 mg	0.52	8.8
2,000 mg	0.32	5.9
**(f) No. 6**		
0 mg	ND	ND
750 mg	0.45	7.8
1,000 mg	0.16	3.0

*(a) Patient No. 1, (b) Patient No. 2, (c) Patient No. 3, (d) Patient No. 4, (e) Patient No. 5, and (f) Patient No. 6*.

**Table 3:** Correlation between changes in urinary free cortisol levels and other parameters.

	Correlation coefficient	*p*-Value
Changes in mean value of salivary cortisol levels	0.75	0.007
Changes in area under the curve of salivary cortisol levels	0.70	0.016
Changes in morning (6:00) serum cortisol levels	0.42	0.200
Changes in morning (6:00) salivary cortisol levels	0.70	0.005
Changes in midnight (22:00) salivary cortisol levels	−0.04	0.915

**Table 4:** Correlation between changes in midnight salivary cortisol levels and changes in mean value (MV) or area under the curve (AUC) of salivary cortisol levels.

	Correlation coefficient	*p*-Value
Changes in AUC of salivary cortisol levels	0.25	0.457
Changes in MV of salivary cortisol levels	0.19	0.582

## Discussion

The present data suggested that multiple measurements of salivary cortisol levels may be useful for optimization of metyrapone treatment by visualization of diurnal cortisol levels. Although multiple collections are required, salivary collection is a less of a burden for patients. Furthermore, salivary samples are stable and can be easily stored (9, 10). Therefore, it is feasible in outpatient clinics.

We clearly demonstrated that measurements of multiple salivary cortisol levels revealed the impaired diurnal cortisol rhythm in patients even after normalization of UFC. The early morning serum cortisol levels and UFC levels remain within the normal range in most patients with subclinical CS (SCS). However, accumulating evidence suggests that the dysregulation of diurnal cortisol rhythms is associated with metabolic comorbidities such as hypertension and impaired glucose metabolism, even in patients with normal UFC levels. A recent systematic review suggested that normalization of the diurnal cortisol rhythms by surgical treatment resulted in an improvement in complications of SCS (14). These data strongly support the clinical importance of not only normalizing the UFC level but also reconstituting in the diurnal cortisol rhythm using multiple salivary cortisol measurements in patients with CS.

In our series, the salivary cortisol levels were constantly elevated throughout the day in patients with cortisol-secreting adrenocortical adenoma, and patients with ACTH-secreting pituitary adenoma had salivary cortisol levels that were elevated mainly in the morning and less in the evening or midnight. These results suggest that the diurnal secretion pattern was relatively preserved in Cushing’s disease as compared with adrenocortical adenomas, indicating that the significance of elevated morning cortisol levels is different depending on the disease etiology.

When determining the timing and dose of metyrapone treatment, it is important to not only normalize the total amount of daily cortisol secretion but also optimize the diurnal rhythm. In this aspect, multiple measurements of salivary cortisol are obviously advantageous for evaluation. Indeed, the prospective case study in Patient No. 4 showed that, although hypercortisolemia was not been controlled despite a high dose (2,250 mg) of metyrapone treatment, the evaluation for diurnal pattern by salivary cortisol levels enabled us to optimize not only the dose but also the timing.

In this study, the salivary cortisol collection was performed five times in a day: at 6:00 (wake up time), 8:00 (before breakfast), 12:00 (before lunch), 18:00 (before dinner), and 22:00 (before sleep). Several centers use the average values of serum cortisol levels from five measurements for evaluation (7, 8). Previous studies have reported that a mean serum cortisol level of 150–300 nmol/L (5–10 µg/dL) is equivalent to a normal production rate (15). However, by this method, blood samples were taken at 9:00, 12:00, 15:00, 18:00, and 21:00, in which it is impractical in outpatient setting. UFC levels have also been used as a monitoring marker (7, 8). However, it is impossible to evaluate diurnal rhythm by the measurement of UFC.

The usefulness of salivary cortisol measurements for the diagnosis and prediction of the risk of the recurrence of CS has been well established (11). However, our data revealed that there was no correlation between changes in UFC levels and those in midnight salivary cortisol levels. In addition, measurements of only midnight salivary cortisol cannot reveal the diurnal rhythm of cortisol because it is affected by the dose and timing of metyrapone administrations. Thus, multiple measurements of salivary cortisol can be more useful than measurements of midnight salivary cortisol.

This study has several limitations. The number of patients was small, especially the preliminary trial in the prospective study. In addition, the effect was evaluated over a short term. The protocol for the dosing and timing was not standardized. Although the symptoms were recorded, the changes in QoL were not evaluated. Thus, further investigation with a large-scale, long-term study is necessary.

In conclusion, although further study is necessary, the present study suggests that taking multiple salivary cortisol measurements could visualize the diurnal change of cortisol during medical treatment and may be a useful for optimizing metyrapone treatment in patients with active CS.

## Ethics Statement

This study was carried out in accordance with the recommendations of the Kobe University Hospital ethical committee with written informed consent from all subjects. All subjects gave written informed consent in accordance with the Declaration of Helsinki. The protocol was approved by the Kobe University Hospital ethical committee.

## Author Contributions

KY drafted the manuscript, and assembled and analyzed the data. HF and YT were responsible for the conception and design of the study. SN, MT, JI, YH, TY, HM, and NS contributed by collecting the data or caring for the patients. The other coauthors contributed to the acquisition, analysis, or interpretation of data for the work. HF was responsible for the critical revision of the manuscript for important intellectual content.

## Conflict of Interest Statement

The authors declare that the research was conducted in the absence of any commercial or financial relationships that could be construed as a potential conflict of interest.
